# Treatment of Rhupus Syndrome With Aplastic Anemia Using Cyclosporine and Hydroxychloroquine: A Case Report

**DOI:** 10.7759/cureus.60875

**Published:** 2024-05-22

**Authors:** Maleeha Saqib, Nighat Shahbaz, Mustafa Malik

**Affiliations:** 1 Internal Medicine, Shifa International Hospital, Islamabad, PAK; 2 Clinical Hematology, National Institute of Bone Marrow Transplant, Rawalpindi, PAK

**Keywords:** aplastic anemia, rhupus, cyclosporine, immunotherapy, cytopenia, rheumatoid arthritis, systemic lupus erythematosus

## Abstract

Rhupus syndrome is an autoimmune disorder that combines the symptoms of lupus and rheumatoid arthritis. It is a rare condition that affects the connective tissues of the body such as the joints, muscles, and skin. The symptoms of rhupus syndrome can be similar to those of lupus, including joint pain, fatigue, and skin rashes. However, rhupus syndrome can also cause symptoms of rheumatoid arthritis, such as joint stiffness and swelling. Treatment for rhupus syndrome usually involves a combination of medications and lifestyle changes to manage symptoms and improve the overall quality of life.

A 24-year-old female patient was referred by a local physician for evaluation of pancytopenia. Her history dates back to six months when she developed progressive fatigue, dyspnea on mild exertion, and polyarthralgia. Initial laboratory investigations revealed pancytopenia, positive antinuclear antibodies (ANA), anti-double-stranded DNA (anti-dsDNA), and anti-cyclic citrullinated peptide (anti-CCP) antibodies. Bone marrow examination confirmed the diagnosis of aplastic anemia. She was started on cyclosporine with an aim to maintain a trough level between 200 and 250 ng/mL. She responded well with hematological recovery in three to four months. This case highlighted the excellent response to cyclosporine hematologically and clinically in rhupus syndrome complicated with aplastic anemia. Further studies are required to establish the long-term efficacy of cyclosporine in this patient population.

## Introduction

Rhupus syndrome is a rare autoimmune overlap syndrome of rheumatoid arthritis (RA) and systemic lupus erythematosus (SLE). Rhupus syndrome is a very rare condition, with only a handful of documented cases in the literature. The prevalence of rhupus syndrome was actually lower (0.09%) than the estimated probability (1.2%) of occurrence. It is associated with immune-mediated clinical and hematological manifestations. It presents with symptoms of RA and SLE although organ damages seen in SLE are not as aggressive in rhupus syndrome [1]. Approximately 250 conditions are grouped under rheumatic diseases which affect various connective tissues of our body and despite the presence of well-defined criteria, it is noted that 25% of people fail to fall under one particular disease. This is where overlap syndromes come into play, of which rhupus syndrome is much discussed and theorized [2].

In this case report, we discuss a 24-year-old female with existing rhupus syndrome, now diagnosed with aplastic anemia. Aplastic anemia in the context of rhupus syndrome presents unique challenges in terms of diagnosis and management. Immunosuppressive therapy including cyclosporine has emerged as a promising option for managing aplastic anemia with concurrent rhupus syndrome.

## Case presentation

**Figure 1:**
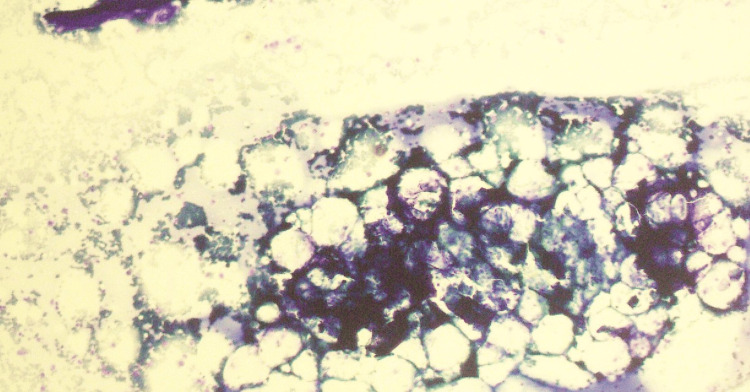
Bone marrow aspirate sample showing a fatty hypocellular marrow with reduced cells from all cell lines consistent with aplastic anemia.

**Figure 2:**
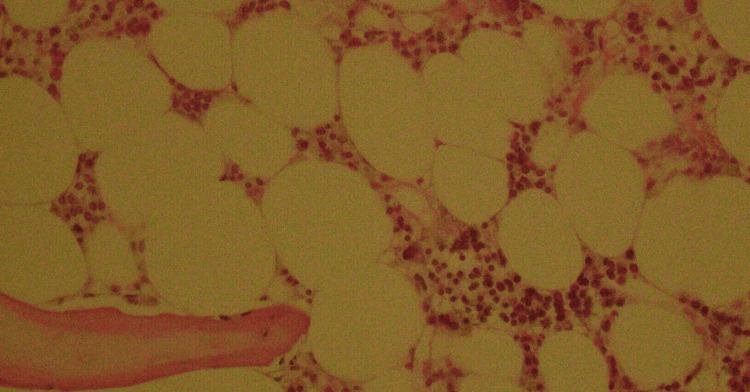
Bone marrow trephine sample of the patient showing a diffusely hypocellular bone marrow. There is trilineage depressed hematopoiesis.

A 24-year-old female was referred by her primary physician for evaluation of pancytopenia. Initially, she reported to a local hospital for generalized bruising, petechiae, and shortness of breath. She had a history of intermittent low-grade fever for the last couple of months, pain in small joints of hands and feet, and a face rash that worsened upon exposure to the sun. On physical examination, a malar rash was noted, and alopecia of 1-2 cm in the right frontoparietal region. There were no swellings or joint deformities on examination and the rest of the systemic examination was unremarkable as well. Her blood counts revealed a white blood count of 2.0x10^9^/L with an absolute neutrophil count of 0.7x10^9^/L, hemoglobin of 4.8 g/dL, and platelets of 20x10^9^/L (Table 1). She was managed with blood components and referred to a tertiary care hospital for further treatment. She underwent an autoimmune workup. The autoimmune profile was positive for anti-nuclear antibody (ANA), anti-double-stranded DNA (anti-dsDNA), and anti-cyclic citrullinated peptide (anti-CCP) antibodies. In light of these results and her pancytopenia, a bone marrow examination was performed, which showed a hypocellular marrow with an overall cellularity of 20%-25% (Figure 1). No abnormal cells or fibrosis were noted on the trephine biopsy examination (Figure 2).

**Table 1:** Lab parameters with reference values.

Parameters	Patient Value on Presentation	Patient Value at 6 months	Reference Range
Hemoglobin (g/dL)	4.8	12.5	12.0-16.0
White blood count (x10^9^/L)	2.0	2.0	4.5-11.0
Absolute Neutrophil count (x10^9^/L)	0.7	2.5	1.6-8.4
Platelets (x10^9^/L)	20.0	215.0	150.0-400.0

Her treatment was initiated with cyclosporine, low-dose steroids, and hydroxychloroquine (HCQ). The initial dose of cyclosporine was 5 mg/kg daily in two divided doses with a target trough level of 200-250 ng/mL. Oral prednisolone was started at 0.5 mg/kg (patient weight was 38 kg) and slowly tapered over the next six months. They were reduced to 5 mg daily at the end of six months, then 5 mg thrice weekly, and were stopped at the end of the first year. Supportive drugs included omeprazole and calcium supplements.

She showed significant hematological and clinical improvement over the next three to four months. At the six-month follow-up, her Hb improved to 12.5 g/dL, platelets 215x10^9^/L, and the absolute neutrophil count had risen to 2.5x10^9^/L. Clinically she was pain-free and attained overall well-being at around six months. Currently, she remains on cyclosporine 50 mg and HCQ 200 mg twice daily. She delivered a healthy baby boy without any maternal or fetal complications on July 12, 2023, i.e., 22 months after starting treatment.

## Discussion

Rhupus syndrome is one of the most infrequent overlap syndromes. There have only been 140 documented cases in literature so far. The first case was described as early as 1960. However, it was not until 1971 that the term rhupus syndrome was coined. This unique syndrome has overlapping features of RA and SLE. It is most commonly characterized by symmetric polyarthritis involving hands, wrists, and knees, malar rash, rheumatoid nodules, and general constitutional symptoms [2,3]. Rhupus syndrome is more commonly seen in women [4,5], often presenting with RA-like symptoms before manifestations of systemic lupus erythematosus.

Diagnosing and differentiating this syndrome from RA or SLE can be challenging. Biomarkers play a key role in this regard, particularly the presence of specific markers like anti-dsDNA and anti-CCP antibodies, which support an overlap of RA and SLE rather than either entity alone [3]. The presence of anti-CCP antibodies may be involved in the pathogenesis of articular erosions. Other biomarkers that may be positive are anti-smith antibodies, anti-SSA, anti-SSB, anti-cardiolipins, rheumatoid factor, and anti-ribonucleoprotein antibodies [6].

Organ involvement like nephritis and neurological manifestations that are common with SLE are not seen as frequently in rhupus syndrome and if present are not as aggressive [2,3]. Peripheral cytopenias are common in RA and SLE but a hypocellular marrow is rarely reported [6]. Autoimmune hemolytic anemia has been reported in rhupus syndrome but the incidence is found very sparingly. Aplastic anemia in rhupus syndrome is mostly due to immune-mediated destruction of bone marrow stem cells leading to the classic hypocellular picture as seen in our patient as well but in general hematological manifestations are uncommon [7].

In our patient, polyarthralgia was the initial presentation. However, there was no evidence of erosive joint arthritis. This was followed by the classic malar rash, which was subsequently followed by pancytopenia. She did not have any organ involvement at the time of presentation or during her treatment. Her autoimmune workup showed a positive ANA, anti-dsDNA, and anti-CCP supporting a diagnosis of overlap syndrome [3].

The best treatment for SLE-associated aplastic anemia has not been defined and different combinations of steroids with immune suppressants have been reported in literature. Immunosuppressive therapy using steroids, HCQ, cyclosporine, intravenous immunoglobulins, anti-thymocyte globulin, and rituximab has been used in various combinations [7,8]. Our patient responded very well to low-dose steroids, cyclosporine and HCQ with complete hematological and clinical remission. Cyclosporine has been associated with a faster recovery of counts and is considered a safe option during pregnancy as well [9,10]. Cyclosporine is an immunomodulatory agent with a major role in suppressing cytokine production involved in T-cell activation. This cytokine suppression is a rationale for its role in combating the aberrant immune response causing aplastic anemia in autoimmune conditions as seen in our patient.

However, there remains a serious lack of properly defined criteria to diagnose rhupus syndrome, which is a challenge during disease classification and subsequent management. This is partly due to a varied clinical presentation and the need for prospective studies with long-term follow-up. They are needed to streamline diagnosis and management [11].

## Conclusions

There is no standard diagnostic and treatment criterion for patients with aplastic anemia with rhupus syndrome which makes the clinical course complicated. Our patient has shown excellent response to cyclosporine. This case report has important implications in clinical practice for treating aplastic anemia in the context of various autoimmune disorders. We recommend multicenter studies to document long-term responses in such cases to establish guidelines that will help in better understanding and recognition of the disease, which will help in early treatment preventing irreversible disease consequences.
